# Preference-Based Serial Decision Dynamics: Your First Sushi Reveals Your Eating Order at the Sushi Table

**DOI:** 10.1371/journal.pone.0096653

**Published:** 2014-05-20

**Authors:** Jaeseung Jeong, Youngmin Oh, Miriam Chun, Jerald D. Kralik

**Affiliations:** 1 Department of Bio and Brain Engineering, Korea Advanced Institute of Science and Technology (KAIST), Daejeon, Republic of Korea; 2 Department of Psychological and Brain Sciences, Dartmouth College, Hanover, New Hampshire, United States of America; Duke University, United States of America

## Abstract

In everyday life, we regularly choose among multiple items serially such as playing music in a playlist or determining priorities in a to-do list. However, our behavioral strategy to determine the order of choice is poorly understood. Here we defined ‘the sushi problem’ as how we serially choose multiple items of different degrees of preference when multiple sequences are possible, and no particular order is necessarily better than another, given that all items will eventually be chosen. In the current study, participants selected seven sushi pieces sequentially at the lunch table, and we examined the relationship between eating order and preference. We found two dominant selection strategies, with one group selecting in order from most to least preferred, and the other doing the opposite, which were significantly different from patterns generated from a random strategy. Interestingly, we found that more females tended to employ the favorite-first rather than favorite-last strategy. These two choice sequences appear to reflect two opposing behavioral strategies that might provide selective advantages in their own right, while also helping to provide solutions to otherwise unconstrained problems.

## Introduction

Imagine that you have just opened a box of assorted chocolates and are deciding on the first pick amongst a diverse and unique collection. A usual favorite, *French Vanilla Truffle*, instantly catches your attention, but then you also see a range of options from caramels to fruits and nuts. In which order do you select? Similar dilemmas arise when determining which songs to listen to in a music player, or whether to hear good news before bad news. Indeed, numerous such situations are encountered daily, given that people have multiple interests, and many of them are experienced serially.

When experiencing a series of events, some evidence suggests that people have a “peak-end bias” in that they prefer the overall experience to end well [1], [2]. Thus, for example, if given a choice between immersing one’s hand into painfully cold water of a constant temperature for 60 sec versus the same cold water for the same 60 sec plus an additional 30 sec, with the temperature remaining painfully cold but gradually increasing, people choose the latter with the longer amount of pain, 90 sec, because the increased temperature at the end results in a better experience overall [2]. This peak-end bias has been found in multiple scenarios, both in the reduction of discomfort and the attainment of positive rewards (*e.g.* receiving one’s favorite candy last), as well as with both ratings of experiences and choices [1]–[3]. Indeed, a peak-end bias appears to be widely reflected in popular culture, from the order of meal entrees (with dessert last) to the climax of performances (such as the choral finale in Beethoven’s Symphony No. 9). Moreover, the peak-end bias appears to reflect a higher-level cognitive ability in humans, both to organize a series of events into a single experience, and to see into the future or mental time travel [4]. Thus, with the peak-end bias, the final event in a sequence–*i.e.* the one furthest into the future–is the most salient.

At the same time, however, other evidence suggests that, like other animals, people discount the value of future events [5]. In fact, it has even been suggested that we may not be too different from other primates when tested with delayed or risky rewards under similar test conditions [6], [7].

Still other evidence suggests that there may be substantial individual differences among people regarding the influence of sequences of events over time. For example, it has been found that individual differences in tests of self-control in childhood (such as waiting for a larger reward versus taking an immediate smaller reward) tend to correlate with measures of executive control and success in adulthood (such as intelligence tests, education level, and socio-economic status [8], [9].

Taken together, it remains unclear whether humans have a preferred sequence order, and whether particular contexts are required for such preferences to manifest themselves. Of particular interest are the many contexts in which individuals directly select the order themselves, such as with meal entrees, or music, reading or to-do lists. Although preference-based decision-making processes have been extensively studied [10]–[13], behavioral strategies for serial decisions has remained poorly understood.

Here, we define ‘the sushi problem’ as a straightforward means to examine how we make serial choices within a given set of items with distinct preference ratings [14]: thus, for instance, how we choose to eat seven sushi pieces at the lunch table. The sushi problem captures essential features of serial choice situations in our daily life; in particular, how to order selections, given that multiple sequences are possible (*e.g.* with seven sushi pieces, there are 5040 possible sequences), and no particular order is necessarily better than another, given that all items will be eaten. How it is answered should provide insight into preference-based serial decision-making and the underlying brain dynamics.

We tested 148 participants (Male : Female = 78∶ 70) between 11 am and 1 pm in randomly-matched groups of two, and we designed a setting in a classroom to emulate a normal dining atmosphere. A main plate with 20 different varieties of sushi was provided (see Fig. S1 in Supplemental Online Material S1), and the participants each chose seven pieces. To determine the degree of preference for seven sushi pieces, the two participants performed seven rounds of rock-paper-scissors. After each game, the winner was allowed to choose a sushi piece first, then the loser picked one of the remaining pieces. This process of playing rock-paper-scissors, then selecting one piece of sushi each, with the winner first and the loser second was repeated seven times until each person had seven pieces. We incorporated the rock-paper-scissors game as a motivational process for selective discrimination because competition under limited resources (in this case, the fixed number of sushi varieties) typically evokes optimal decision-making to maximize utility [15]. In line with our assumption, the participants verified at the end of the experiment that they were indeed selecting in order of preference and were eager to win the rock-paper-scissors game to choose their favorites. The participants were then allowed to eat their sushi once all seven pieces were picked, which was monitored using camcorders. Eating behavior the eating order was compared with the order of selection (i.e., the degree of preference).

## Materials and Methods

### Experimental Procedures

Two participants constituted a pair competing at a sushi table to determine who would make the first sushi selection. The setting of the sushi table is displayed in Fig. S1 in Supplemental Online Material S1). The entire procedure of sushi selection and eating was recorded by web cameras. For each subject, the first sushi piece selected was denoted as “Preference 1”, the second as “Preference 2”, and so on. Additionally, the order that subjects ate the sushi was recorded by denoting the first sushi piece eaten as “Eating order 1”, the second as “Eating order 2”, and so on. Preference and eating order for each sushi piece were recorded together; for example, if a subject ate the second-selected sushi first, then this event was labeled as “Preference 2– Eating order 1”. For each subject, seven events were recorded in this manner. Thus, this record shows the relationship between selection order and eating order.

After the experiment was completed, the subjects were asked to answer survey questions about their education level, monthly expenditure, annual familial income, sibling relationship, and frequency of sushi eating to investigate possible relationships with the pattern of sushi eating observed in the study. All experimental processes were video-recorded throughout the experiment. The Institutional Review Board (IRB) of KAIST approved all experimental procedures for this study. The participants provided their written informed consent to participate in this study.

### Subject Number and Saliency Matrices

From the preference-eating order record for all 148 subjects, we built a 7×7 subject number matrix by counting the number of subjects corresponding to each element of the matrix. The horizontal and vertical axes represented eating order and preference, respectively. To identify matrix elements whose values were significantly larger than randomly expected values, we constructed a random matrix built from 1,000 random eating sequences in a fashion similar to that used to construct the subject number matrix. By making 1,000 such random matrices, we obtained a z-matrix whose elements consisted of z-scores defined by the following equation:
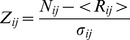
(1)where *Z_ij_*, *N_ij_*, <*R_ij_*>, and *σ_ij_* indicate each element of the z-matrix, the subject matrix, the mean value of 1,000 random matrices, and the standard deviation of the random matrices, respectively. Assuming the null hypothesis that these z-scores follow the normal distribution, we generated saliency matrices whose elements were 1−*p*, where *p* is the *p*-value for obtaining such large numbers by chance alone, and we identified elements with significantly larger values. In addition to these individual element tests, we also applied Chi-square test to the whole subject number matrix and random matrices to see whether eating order and degree of preference are independent with each other. The slope of empirical data obtained from the subject subgroup and the average slope of 1,000 random sequences of each subject in a subgroup were compared by Wilcoxon ranksum test.

### Subject Group Classification

Group classification was made on the basis of the preference of the first-eaten sushi. For example, Group 1 consisted of subjects who ate their favorite sushi (Preference 1) first; Group 2 consisted of those who ate their second-favorite sushi (Preference 2) first, and so on. We counted the number of subjects in each group and obtained a distribution of subjects. Additionally, we performed the same z-score analysis as described above, however, in this case, for each subject group to determine whether the first choice influenced the following series of selection behaviors. To guarantee a fair comparison, each random matrix was built from the same defining conditions used for the corresponding subject group. For example, the random matrix that corresponded to Group 3 had the same restriction as the original subject group, i.e., that the third-favorite sushi was selected first.

In addition, for each subject group and its corresponding random group, we obtained the average slope of the preference and eating order relationship. We first calculated the linear regression slope for each subject or random sequence in individual ‘preference–eating order’ space, and then we measured the mean and standard error of this slope for each group. The purpose of this slope calculation was to determine whether each subject in a group indeed followed the overall eating pattern of that group.

## Results

### Subject Number and Saliency Matrices

**Figure 1:**
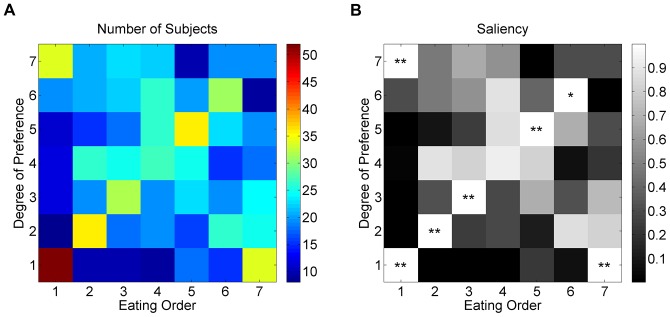
Relationship between eating order and the degree of preference. Subject number matrix (A) and Saliency matrix (B). The horizontal axis represents the eating order, and the vertical axis represents the degree of preference. Each element of the matrix represents (A) the number of subjects making the particular choice and (B) 1– *p*, where *p* is the probability that the number of subjects in the matrix cell resulted from random order selections; *p<0.05; **p<0.01.

The subject number matrix for all 148 subjects is shown in Fig. 1A. Each element of the matrix represents the number of subjects matched to the specific preference and eating order pair. For example, the bottom-left cell represents the number of subjects who ate their most preferred sushi piece first (N = 52). Fig. 1B shows the degree to which the matrix elements were larger than the corresponding matrix elements generated from random sequences. Both matrices show a significantly high concentration of subjects along the bottom-left to top-right diagonal elements, combined with matrix elements (1,7) and (7,1). This finding suggests a mixture of two distinct eating patterns: one in which the sushi is eaten in order of preference (diagonal), and the other following the opposite pattern, in which the least preferred is eaten first, and the best is saved for last (anti-diagonal). In addition, chi-square test on the subject number matrix verified that there is a strong relationship between eating order and degree of preference 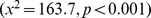. On the other hand, random matrices showed independence between the two variables on average 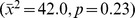.

### Subject Group Classification

**Figure 2:**
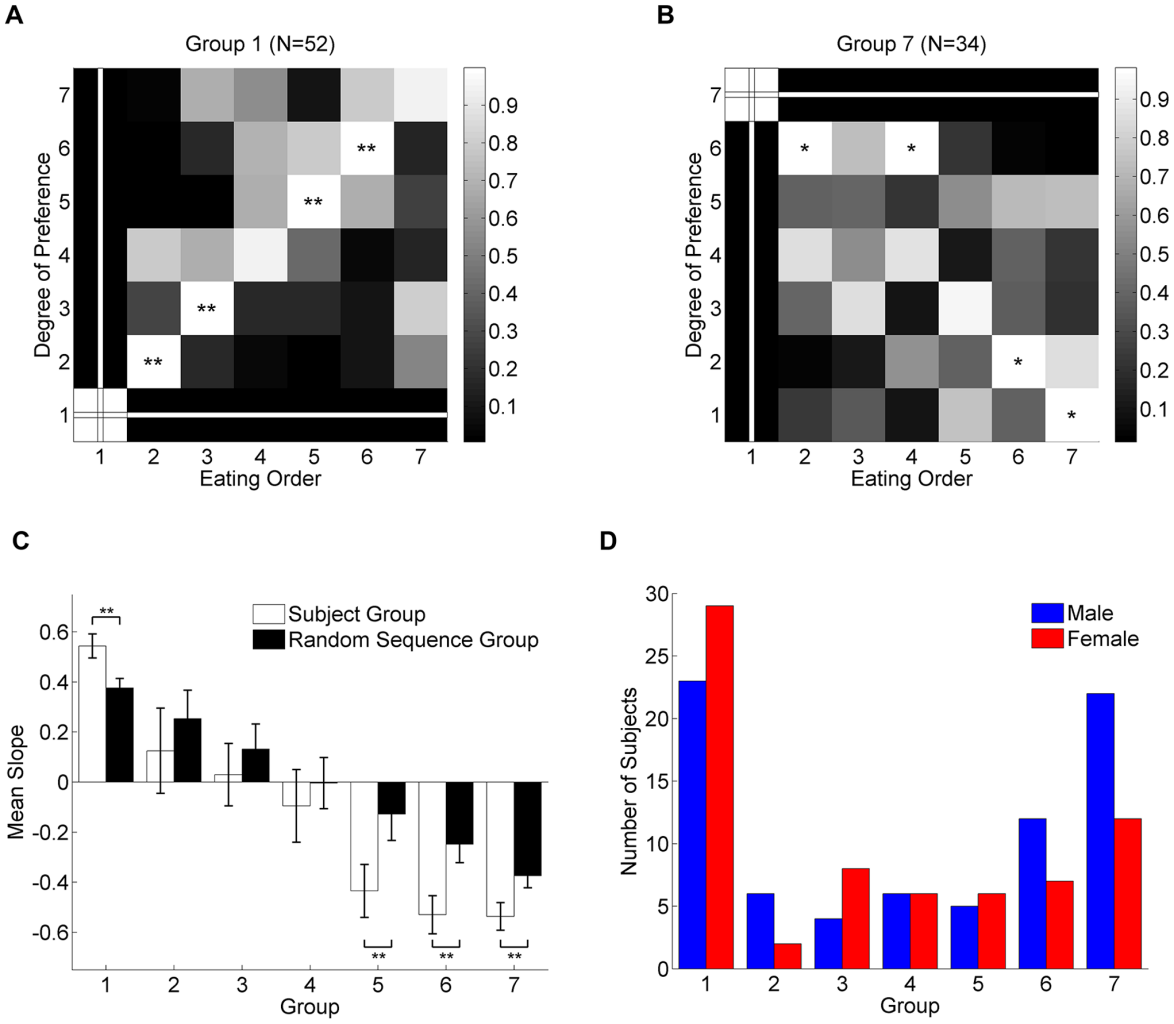
Comparison of saliency maps with random sequences. (A–B) Saliency maps for Groups 1 and 7. Each element of the matrix represents 1– *p*, where *p* is the probability that the number of subjects derived from random order selections; *p<0.05; **p<0.01. Thin white lines demarcate the confining condition for each group; no subject can be located in such areas. (C). Mean slope of the linear regression relating eating order to preference. The slope of Group 1 is significantly higher than that of its random pair, whereas Groups 5, 6, and 7 show significantly lower slopes (**p<0.01). (D). Number of participants in each group. The Kolmogorov-Smirnov test was applied to test for an uneven distribution, *p*<0.01 (male), *p*<0.001 (female).

Next, we divided subjects into seven groups according to the preference ranking of the sushi eaten first. For example, Group 3 consisted of the subjects who ate their third-favorite sushi first. Again, we found that the majority of subjects chose to eat either the most preferred sushi (Group 1, N = 52) or the least preferred one (Group 7, N = 34) first (Figure 2A and 2B, Table 1).

**Table 1:** Participant numbers in each group.

Group	Male	Female	Total
1	23	29	52
2	6	2	8
3	4	8	12
4	6	6	12
5	5	6	11
6	12	7	19
7	22	12	34
Total	78	70	148
*p*-value	0.002	0.000212	1.08E-06

*p*-values obtained from a test to determine whether the distribution was uneven (Kolmogorov-Smirnov test) are also provided.

The remaining groups had 8–12 members each (except for Group 6; with 19 subjects, this group was still smaller than Group 1 or 7 but larger than the other groups) (Kolmogorov-Smirnov, see Table 1). This polarized distribution of subjects indicates again that two opposing eating behaviors were the dominant behavioral strategies. Interestingly, female and male subjects showed different distributions among groups: while males were symmetrically distributed between Groups 1 and 7, females were more prominently skewed toward Group 1 (Fig. 2D and Table 1).

### Preference and Eating Order for Subject Groups

To further characterize the relationship between the initial choosing order (*i.e.* preference) and eating order, we examined whether the first-eaten sushi predicted the following series of selections. Saliency maps and the results of the z-score analysis performed for the seven subject groups are shown in Fig. 2A and 2B (Group 1 and Group 7) and Fig. S2 in Supplemental Online Material S1 (other groups in Supplemental Online Material S1). Thin white lines indicate areas in which no subjects can theoretically be found because each random matrix was obtained using the same conditions as the corresponding subject group. For example, the random sequences corresponding to Group 1 all start with 1. This satisfies the condition “eating the most favorite sushi first”, and thus the element (1,1) of the matrix is completely filled and all other elements of row 1 and column 1 are empty. Group 1 shows prominent diagonal elements, whereas Group 7 (as well as Groups 5 and 6) exhibited nearly anti-diagonal (i.e., negatively sloped) elements. The diagonal concentration of subject numbers in Group 1 implies that these subjects tended to eat in the order of descending preference, eating their favorite sushi first before less-favored varieties. Similarly, the anti-diagonal elements of Groups 5, 6, and 7 imply that subjects of these groups usually ate in the order of ascending preference, starting with their least-favorite first while saving their favorites for last.

To assess whether individuals in each subject group indeed followed the general eating patterns of that group, we calculated the average slope for each group. Slopes were obtained from a ‘preference-eating order’ graph individually generated for each subject using a linear regression model. The average slope of Group 1 was significantly higher than that of the corresponding random sequence group, whereas those of Groups 5 to 7 were significantly lower (Fig. 2C). However, the average slope of other variables (such as a monthly expenditure, annual family income, number of siblings etc.) did not show any significance (Table S1 to S8 in Supplemental Online Material S1, Fig. S2 to S9 in Supplemental Online Material S1).

## Discussion

This straightforward sushi serial-choice paradigm provides a means to examine how we order our choices when selecting among multiple items sequentially without replacement: *i.e.* one-by-one until all items are selected. Serial choice sequences occur throughout daily life, from the meals we eat to the order of our daily activities. After ‘winning’ their more-preferred sushi pieces, people were then allowed to eat them in any order they wanted. The ‘sushi problem’ faced is how to order the selections, given that multiple sequences are possible (*e.g.* with seven items, there are 5040 possible sequences), and no particular order is necessarily better than another, given that all items will be eaten. If, however, highly structured and common choice patterns were found, it would provide insight into how decision-making mechanisms in the brain impose constraints on an otherwise under-constrained problem.

In the experiment, we found a significant correlation between the degree of preference and eating order. More specifically, although multiple sequences were possible, we generally found a bimodal distribution of people using one of two dominant sequential-choice strategies: eating their favorite sushi first or saving them for last.

The most popular strategy was eating in order of preference. This finding aligns with those that show that people, like nonhuman animals, generally discount future events [6], [7]. Such a strategy might reflect relative impulsiveness [8], [9]. However, this valuation and decision-making process might in fact be optimal in complex, uncertain, and competitive environments [5], [15]. It is in fact possible that the uncertain and competitive nature of the experimental setting might itself have contributed to the employment of this strategy. However, the specific testing paradigm used here, in which all items were certain and would be eaten (*i.e.* serial choice without replacement), cannot explain why individuals would employ the best-first strategy. Thus, we conclude that some people appear to follow the strategy independent of context at least to some degree.

The second most popular strategy found in the current experiment was eating in the opposite order of preference: *i.e.* saving the best for last. In fact, other evidence has shown that people often exhibit a peak-end bias with event sequences, preferring those that finish on the highest note–at the peak [1], [2]. This peak-end bias might reflect a heightened cognitive ability to organize events into sequences, as well as to increase the impact of future events, at the expense of more immediate ones. In so doing, the bias might lead to heightened impulse control and more accurate future predictions (to the extent that newer information at the end of a sequence is more accurate than older). Indeed, numerous aphorisms in popular culture such as “saving the best for last” or “all’s well that ends well” appear to reflect the value of this behavioral strategy.

Given the mixture of findings in the literature, it may not be surprising to find significant individual differences [1], [8], [9]. However, although there were cases in which other strategies were employed, two proved to be dominant. These two general strategies might reflect a stable state in which two opposing strategies provide selective advantages in their own right [8], [16]. Whether these behavioral strategies reflect selective adaptations resulting in inherent personality dispositions or learned behavioral patterns is yet to be determined [8], [9].

Either way, if the employment of either of these two strategies reflects a broader behavioral pattern, we might expect correlations with other related traits such as education or income level [8], [9]. Although we did not find correlations with such variables in the current study, our sample population may not have provided a wide enough range of values. For example, all participants were current university undergraduates or held undergraduate degrees. Thus, more detailed work is necessary to determine the stability and influence of these behavioral strategies in other behavioral contexts. In the study, we did, however, obtain a difference between males and females, in that more females tended to employ the favorite-first rather than favorite-last strategy. At this point, it is unclear why this gender difference was found. For example, the females may have considered the setting to be more uncertain, competitive, and risky, which could have promoted a more choose-the-best-first strategy [8].

In general, it is clear that further work will need to delineate the conditions under which these behavioral strategies are employed. For example, it is likely that most people prefer a peak-end sequence in some cases (such as the climax of stories or performances) [1], [2]. In contrast, in other scenarios, such as competitive ones, a peak-first preference might be optimal [5], [15]. Indeed, the specific behavioral paradigm itself might also influence sequence preferences, such as serial choices versus the experience of a sequence of events independent of behavior (e.g. watching performances) [17].

As a consequence of exhibiting these two general sequential-choice strategies in the current study, we found that the entire eating sequence could generally be determined by the first selection, at least for those who selected their favorite (Group 1) or least favorites (Groups 5–7) first, which were the majority of participants (78%). Thus, our results support the contention that relatively simple diagnostic tests can uncover general cognitive and behavioral strategies of individuals, which would reflect the relative influence of different underlying brain circuits [8], [9], [18], [19]. For serial choice, in particular, it will be important to determine the extent that these two dominant strategies that we have uncovered are employed in other contexts to determine how much predictive power is achieved from the observation of one’s first sushi selection.

Our finding should be interpreted with caution, because the ascending choice sequence, by definition, starts with the most preferred sushi piece, whereas the descending sequence starts with the least preferred one as shown in Fig. 2C whose heights decrease with the group index. To overcome this circularity issue, we used the random sequences to find any significant difference between the random sequence group and the subject group, yet this is also possible to be ascribed to the sample bias due to the small number of random matrices or the small number of the subject data in group 5 and 6. Thus, similar experiments with a large number of subjects should be performed to support this finding in future.

## Supporting Information

Supplemental Online Material S1 **Figure S1,** The sushi table setting. **Figure S2,** A–E: Saliency matrices for Groups 2 to 6. White elements crossed by thin white lines indicate the confining conditions for each group (*i.e.*, all subjects in each group must be contained in the matrix element at the intersection of the two lines). **Figure S3,** A: Finger length ratio for males and females. B: Finger length ratio for each group. C: Finger length ratio and linear slope of eating order for each individual. **Figure S4,** Education level and mean linear slope of eating order. **Figure S5,** Monthly expenditure and mean linear slope of eating order. **Figure S6,** Annual family income and mean linear slope of eating order. **Figure S7–S8,** Sibling relationship and mean linear slope of eating order. **Figure S9,** Frequency of sushi eating and mean linear slope of eating order. **Table S1,** Finger length ratio for males and females. **Table S2,** Finger length ratio for each group. **Table S3,** Education level. **Table S4,** Monthly Expenditure. **Table S5,** Annual Income of Family. **Table S6,** Number of Siblings. **Table S7,** Order in Siblings. **Table S8,** Frequency of Sushi Eating.
(DOC)
