# Supplementary material for: Preference-Based Serial Decision Dynamics: Your First Sushi Reveals Your Eating Order at the Sushi Table
Source: PLoS One. 2014 May 20;9(5):e96653. doi: 10.1371/journal.pone.0096653 (PMC4028175; doi:10.1371/journal.pone.0096653)
Supplement: Supplemental Online Material S1 — Figure S1, The sushi table setting. Figure S2, A–E: Saliency matrices for Groups 2 to 6. White elements crossed by thin white lines indicate the confining conditions for each group (i.e., all subjects in each group must be contained in the matrix element at the intersection of the two lines). Figure S3, A: Finger length ratio for males and females. B: Finger length ratio for each group. C: Finger length ratio and linear slope of eating order for each individual. Figure S4, Education level and mean linear slope of eating order. Figure S5, Monthly expenditure and mean linear slope of eating order. Figure S6, Annual family income and mean linear slope of eating order. Figure S7–S8, Sibling relationship and mean linear slope of eating order. Figure S9, Frequency of sushi eating and mean linear slope of eating order. Table S1, Finger length ratio for males and females. Table S2, Finger length ratio for each group. Table S3, Education level. Table S4, Monthly Expenditure. Table S5, Annual Income of Family. Table S6, Number of Siblings. Table S7, Order in Siblings. Table S8, Frequency of Sushi Eating. (DOC) [file pone.0096653.s001.doc]

**Supplemental Online Material**

**Preference-based serial decison dynamics:**

**Your first sushi reveals your eating order at the sushi table**

**Jaeseung Jeong, Youngmin Oh, Miriam Chun, Jerald D. Kralik**

**Results**

**Finger length ratio for each group**

Since it is widely known that the finger length ratio (the second digit to the fourth digit) and sexual or certain behavioral patterns are related due to the influence of exposure to prenatal androgen (Williams et al., 2000; Bailey & Hurd, 2005), we recorded the finger lengths of the right hands of subjects using a photocopying machine. We investigated the possible influence of this factor on eating behavior. The 2D/4D finger length ratio was averaged for male and female subjects as well as within each group.

**Relationship to the 2D/4D finger length ratio**

Although the 2D/4D finger length ratio was significantly different between males and females as shown in previous studies [Student’s *t-test*, *p*=0.01, Table 2, Fig. S3A], no evident difference in the finger length ratio was found among different subject groups [ANOVA, Table 3, Fig. S3B]. The results were also not significant for groups containing only male or female subjects. One point to note, however, is the difference in the finger length ratio between males and females in each group. Only in Group 1 did males and females exhibit different finger length ratios; although this difference between genders exists for the total population, no other groups showed a significant difference. Further, the finger length ratio and linear slope of the eating order showed no significant correlation (Fig. S3C).

**References**

Williams, T.J., Pepitone, M.E., Christensen, S.E. et al. (2000). Finger-length ratios and sexual orientation. *Nature* 404, 455–456.

Bailey, A.A., & Hurd, P.L. (2005). Finger length ratio (2D:4D) correlates with physical aggression in men but not in women. *Biological Psychology* 68, 215–22.

**Tables**

**Table S1**. Finger length ratio for males and females

| Group | 2D/4D | Std |
| --- | --- | --- |
| Male | 0.939 | 0.036 |
| Female | 0.956 | 0.040 |
| p-value | 0.01 | ∙ |

**Table S2**. Finger length ratio for each group

|  | Total | | Male | | Female | |
| --- | --- | --- | --- | --- | --- | --- |
| Group | Mean | Std | Mean | Std | Mean | Std |
| 1 | 0.943427 | 0.035553 | 0.928057 | 0.029158 | 0.952847 | 0.036238 |
| 2 | 0.945879 | 0.057912 | 0.036238 | 0.043038 | 0.956319 | 0.111395 |
| 3 | 0.951998 | 0.040149 | 0.940084 | 0.046306 | 0.957956 | 0.03992 |
| 4 | 0.964969 | 0.048734 | 0.958154 | 0.037563 | 0.971783 | 0.060848 |
| 5 | 0.927852 | 0.040961 | 0.912765 | 0.044089 | 0.946711 | 0.032075 |
| 6 | 0.9574 | 0.026055 | 0.954731 | 0.028033 | 0.961848 | 0.024167 |
| 7 | 0.94727 | 0.041042 | 0.941282 | 0.03891 | 0.957249 | 0.044251 |
| p-value | 0.393 | ∙ | 0.237 | ∙ | 0.967 | ∙ |
| F | 0.055 | ∙ | 1.379 | ∙ | 0.226 | ∙ |

**Table S3**. Education level

| Undergraduate Student | University Graduate | Above Graduate School |
| --- | --- | --- |
| 122 | 13 | 8 |

**Table S4**. Monthly Expenditure

| Below $300 | $301~$600 | $601~$1,000 | Over $1,000 |
| --- | --- | --- | --- |
| 81 | 54 | 6 | 2 |

**Table S5. Annual** Income of Family

| Less than $10,000 | $10,000 ~ $30,000 | $30,001 ~ $50,000 | $50,001 ~ $100,000 | Over $100,000 |
| --- | --- | --- | --- | --- |
| 12 | 49 | 56 | 22 | 4 |

**Table S6**. Number of Siblings

| One | Two | Three | Four | Five | Six |
| --- | --- | --- | --- | --- | --- |
| 10 | 89 | 28 | 13 | 2 | 1 |

**Table S7**. Order in Siblings

| First | Second | Third | Fourth | Fifth | Sixth |
| --- | --- | --- | --- | --- | --- |
| 66 | 60 | 8 | 7 | 1 | 1 |

**Table S8**. Frequency of Sushi Eating

| Every day | 2~3 times a week | once a week | once a month | once a 2~3 months | Seldom |
| --- | --- | --- | --- | --- | --- |
| 0 | 1 | 5 | 60 | 52 | 25 |

**Figure Legends in Supplemental Online Material**

**Figure S1.** The sushi table setting

**Figure S2.** A-E: Saliency matrices for Groups 2 to 6. White elements crossed by thin white lines indicate the confining conditions for each group (*i.e.*, all subjects in each group must be contained in the matrix element at the intersection of the two lines).

**Figure S3.** A: Finger length ratio for males and females. B: Finger length ratio for each group. C: Finger length ratio and linear slope of eating order for each individual.

**Figure S4***.* Education level and mean linear slope of eating order.

**Figure S5***.* Monthly expenditure and mean linear slope of eating order.

**Figure S6.** Annual family income and mean linear slope of eating order.

**Figure S7-S8***.* Sibling relationship and mean linear slope of eating order.

**Figure S9.** Frequency of sushi eating and mean linear slope of eating order.


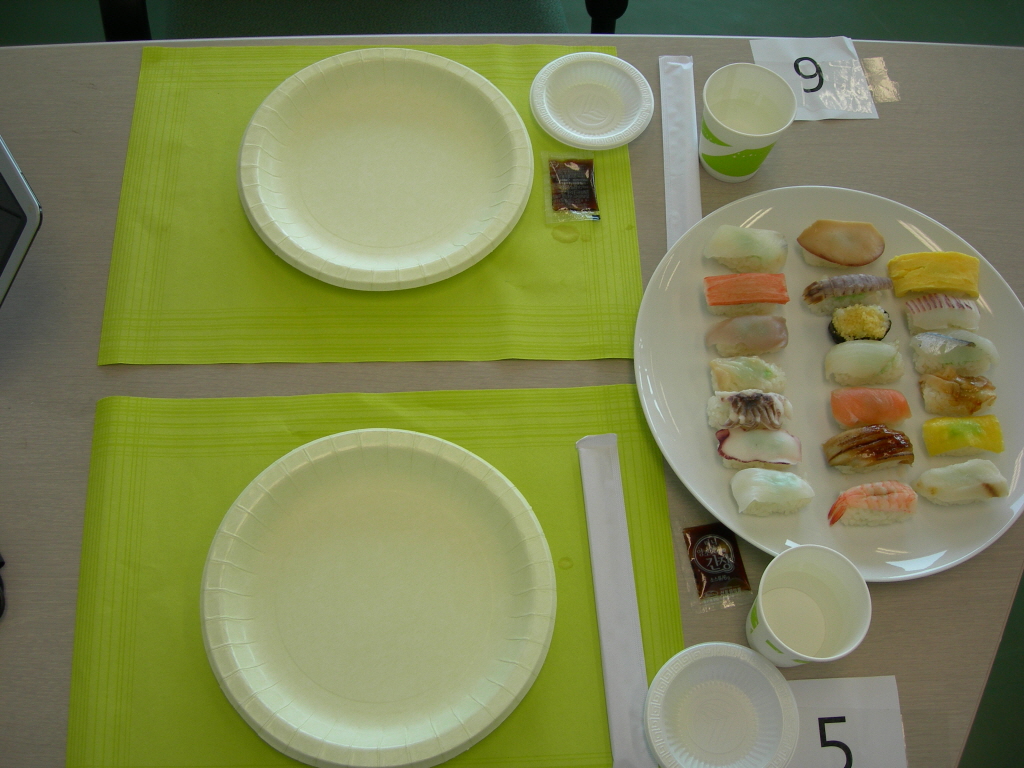


**Figure S1. Jeong et al.**

**
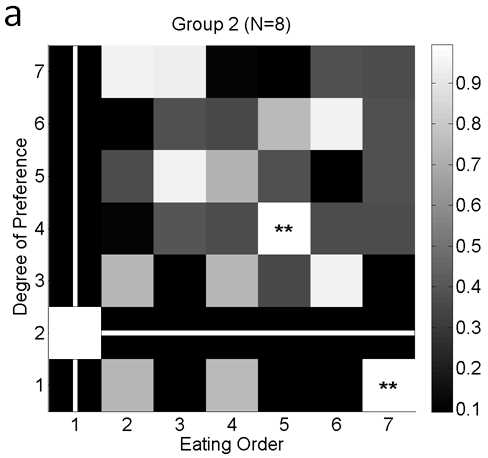

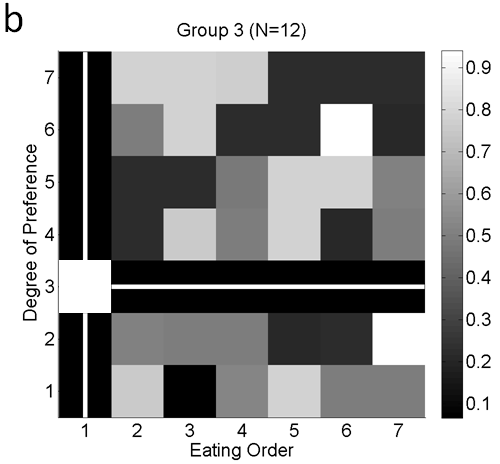

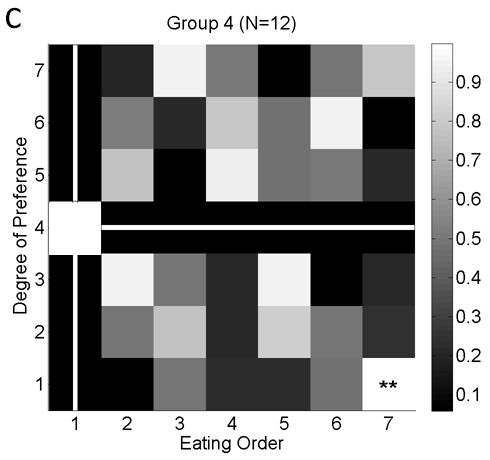

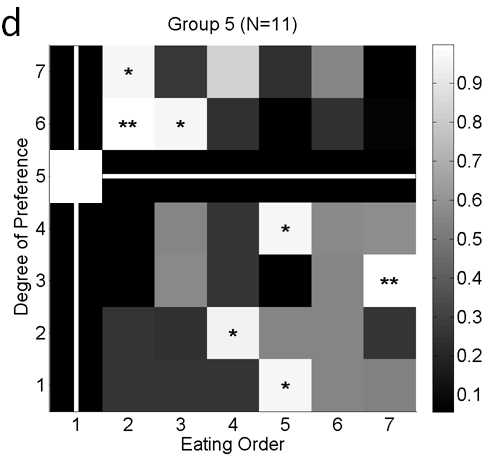

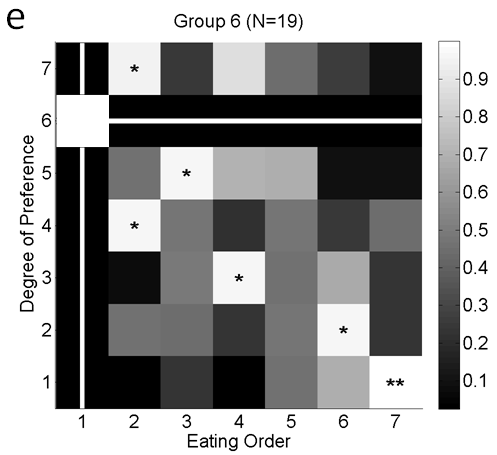
**

**Figure S2. Jeong et al.**


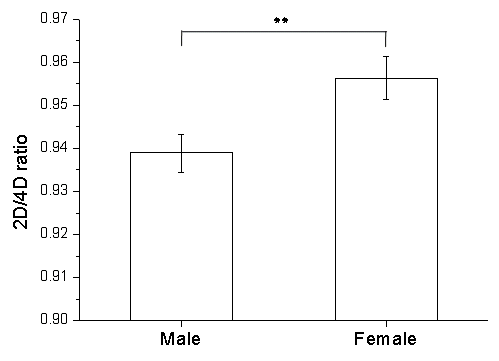
**a**


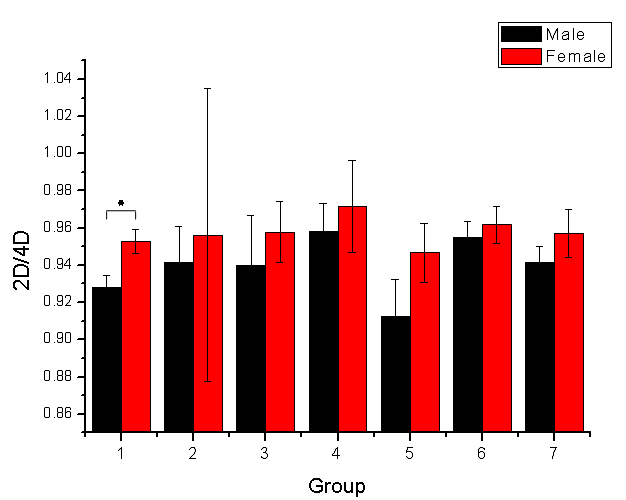


**b**

**C**

**Figure S3. Jeong et al.**

**Figure S4. Jeong et al.**

**Figure S5. Jeong et al.**

**Figure S6. Jeong et al.**

**Figure S7. Jeong et al.**

**Figure S8. Jeong et al.**

**Figure S9. Jeong et al.**
